# Impact of the volume of the myelomeningocele sac on imaging, prenatal neurosurgery and motor outcomes: a retrospective cohort study

**DOI:** 10.1038/s41598-021-92739-2

**Published:** 2021-06-23

**Authors:** Romain Corroenne, Amy R. Mehollin-Ray, Rebecca M. Johnson, William E. Whitehead, Jimmy Espinoza, Jonathan Castillo, Heidi Castillo, Gunes Orman, Roopali Donepudi, Thierry A. G. M. Huisman, Ahmed A. Nassr, Michael A. Belfort, Magdalena Sanz Cortes, Alireza A. Shamshirsaz

**Affiliations:** 1 Department of Obstetrics and Gynecology, Texas Children’s Hospital & Baylor College of Medicine, Houston, TX USA; 2 E. B. Singleton Department of Pediatric Radiology, Texas Children’s Hospital, Houston, TX USA; 3 Department of Radiology, Baylor College of Medicine, Houston, TX USA; 4 Department of Neurosurgery, Texas Children’s Hospital & Baylor College of Medicine, Houston, TX USA; 5 Department of Pediatrics, Texas Children’s Hospital & Baylor College of Medicine, Houston, TX USA; 6 Division of Fetal Therapy and Surgery, Baylor College of Medicine, Houston, TX 77030 USA

**Keywords:** Outcomes research, Paediatric research

## Abstract

To investigate the association of the myelomeningocele (MMC) volume with prenatal and postnatal motor function (MF) in cases who underwent a prenatal repair. Retrospective cohort study (11/2011 to 03/2019) of 63 patients who underwent a prenatal MMC repair (37 fetoscopic, 26 open-hysterotomy). At referral, measurements of the volume of MMC was performed based on ultrasound scans. A large MMC was defined as greater than the optimal volume threshold (ROC analysis) for the prediction of intact MF at referral (2.7 cc). Prenatal or postnatal intact motor function (S1) was defined as the observation of plantar flexion of the ankle based on ultrasound scan or postnatal examination. 23/63 participants presented a large MMC. Large MMC lesions was associated with an increased risk of having clubfeet by 9.5 times (CI%95[2.1–41.8], p < 0.01), and reduces the chances of having an intact MF at referral by 0.19 times (CI%95[0.1–0.6], p < 0.01). At birth, a large MMC reduces the chance of having an intact MF by 0.09 times (CI%95[0.01–0.49], p < 0.01), and increases the risk of having clubfeet by 3.7 times (CI%95[0.8–18.3], p = 0.11). A lower proportion of intact MF and a higher proportion of clubfeet pre- or postnatally were observed in cases with a large MMC sac who underwent a prenatal repair.

**Trial registration:** Clinicaltrials.gov NCT02230072 and NCT03794011 registered on September 3rd, 2014 and January 4th, 2019.

## Introduction

In the United States, myelomeningocele (MMC), a type of open neural tube defect (NTD), is seen in 1 in 3000 live-births^1^. Myelomeningocele occurs when the neural placode extends through the spinal defect with accompanying herniation of the meninges. Exposure of the neural elements to amniotic fluid causes progressive injury, which leads to changes in the cytoarchitecture of the spinal cord from irritation of the primary lesion^2–7^. Because of this, motor and sensory deficits accompany the diagnosis of MMC^2–5, 8^. The Management of Myelomeningocele Study (MOMS), a multicenter randomized clinical trial, reported improved motor function and a decrease in the need for ventriculo-peritoneal shunting during the first 12 months of life in those patients who underwent prenatal MMC repair as compared to the postnatal repair patients^9^.

Oliver et al. demonstrated that MMCs prenatally or postnatally repaired were associated with a higher rate of clubfeet and lower extremity impairment when compared to myeloschisis lesions, another type of open NTD where the spinal cord remains within the spinal canal^10^. They hypothesized that additional neurologic injury may result from cerebrospinal fluid hydrostatic forces within the MMC sac with secondary stretching of the neural placode and spinal nerves, which would represent another acquired mechanism of injury in cases of open spinal dysraphism.

In large MMCs, the excessive posterior herniation of the neural placode outside of the level of the wide open osseous spinal canal results in more significant stretching and thinning of the neural placode as well as the spinal nerves that are located along the anterior surface of the neural placode. We hypothesized that the overstretching of the neural placode in cases with large MMC volumes will result in higher motor function impairment.

The objectives of our study were: (1) to compare brain findings between large and non-large MMC sac volumes at the time of referral and 6 weeks after the prenatal surgery; (2) to compare prenatal motor function from mid-gestation to delivery between large and non-large MMC sac volume; and (3) to determine the relationship between the volume of the lesions and postnatal neurological short-term outcomes.

## Results

### Characteristics of the population

Sixty-three MMC cases who underwent prenatal NTD repair were included in this study (37 fetoscopic and 26 open-hysterotomy). The median volume of the sac at the time of referral was 1.6 (0.05–44.5) cc. Twenty-three cases presented with a “large” sac and 40 with a “non-large” sac.

### Volume of the lesion and the neuroanatomic findings by MRI at the time of referral and 6 weeks after the prenatal surgery

At the time of referral, the anatomical level of the lesion and ventricle size were similar between large and non-large MMC lesions (Table 1). Six weeks after the surgery, there was no difference between large and non-large MMC sacs for the size of the ventricles and the proportion of fetuses that showed hindbrain herniation reversal (Table 1).

**Table 1:** Comparison of neurological, neurosurgical and neonatal characteristics between large and non-large myelomeningocele in the entire cohort.

	Large lesions (n = 23)	Non-large lesions (n = 40)	p^a^
Maternal age (years)	27.4 ± 5.7	30.1 ± 6.5	0.10
Body mass index	25.8 ± 4.6	26.8 ± 4.3	0.40
Race (white)	22/23 (96)	36/40 (90)	0.64
Ethnicity (hispanic)	6/23 (26)	8/40 (20)	0.58
**Pre-operative MRI evaluation**			
Gestational age at the time of referral (weeks)	23.4 (19–25.5)	23 (18–25.7)	0.42
Volume of the lesion (cc)	4.5 (3–44.5)	1 (0.05–2.4)	< 0.01
Anatomical level of the lesion ≥ L2 (%)	7/23 (30)	8/40 (20)	0.35
Mean size of bilateral posterior ventricular horns at referral (mm)	11.5 (7–18)	12 (6–18)	0.81
Ventriculomegaly defined as mean of posterior horn width > 10 mm (%)	16/23 (70)	26/40 (65)	0.71
Severe ventriculomegaly defined as mean of posterior horn width > 15 mm (%)	5/23 (22)	9/40 (22)	0.94
Clubfeet at referral (at least one foot, %)	13/23 (56)	4/40 (10)	< 0.01
**Pre-operative US motor function evaluation**			
Intact motor function (first sacral level) at the time of referral (%)	12/23 (52)	33/40 (17)	< 0.01
Motor function at the time of referral (metameric level)	L4 (L1-S1)	S1 (L1-S1)	< 0.01
Gestational age at the time of pre-operative US motor function evaluation (weeks)	23.4 (18.7–25.5)	23.0 (18–25.7)	0.41
**Surgery**			
Gestation age at the time of surgery (weeks)	25.3 (24–25.9)	24.6 (21.3–26)	< 0.01
Interval between time of pre-operative MRI and surgery (weeks)	1.5 (0.1–7)	1.4 (0.1–7.9)	0.72
Fetoscopic repair technique (%)	14/23 (69)	23/40 (55.3)	0.79
Duration of surgery (min)	250 (129–394)	202 (94–356)	0.18
Need for relaxing incisions (%)	3/23 (13)	4/40 (10)	0.70
**Post-operative MRI evaluation**			
Gestational age of post-operative MRI (weeks)	30.6 (28–33)	30.4 (27–35)	0.37
Hindbrain herniation severity^b^	1 (0–3)	1 (0–3)	0.97
Reversal of hindbrain herniation after surgery (%)	13/19 (68)	28/35 (80)	0.34
Ventricle size after surgery (mm)	15.5 (10–26.5)	16 (6–23)	0.94
Ventriculomegaly defined as mean of posterior horn width > 10 mm (%)	19/19 (100)	32/37 (86)	0.15
Severe ventriculomegaly defined as mean of posterior horn width > 15 mm (%)	10/19 (53)	24/37 (65)	0.37
**Post-operative US motor function evaluation**			
Gestational age at the time of US motor function evaluation 6 weeks after the surgery (weeks)	30.6 (28.5–33)	30.4 (26.2–35)	0.92
Intact motor function 6 weeks after the surgery (%)	9/19 (47)	24/35 (69)	0.92
Motor function 6 weeks after the surgery (metameric level)	L4 (L1-S1)	S1 (L1-S1)	0.14
Gestational age at the time of the last US motor function evaluation before delivery (weeks)	36.0 (32–39)	34.9 (30–40)	0.18
Intact motor function at last US scan before delivery (%)	5/12 (42)	19/24 (79)	0.02
Motor function at last US scan before delivery (metameric level)	L4 (L1-S1)	S1 (L1-S1)	0.04
**Neonatal outcomes**			
Gestational age at delivery (weeks)	36 (26–40)	36.3 (27.3–40.6)	0.87
Female gender (%)	7/23 (30)	21/40 (52)	0.09
Birth weight (grams)	2575 (870–3745)	2665 (624–4430)	0.55
Dehiscence or leakage of CSF at birth (%)	2/23 (9)	10/40 (25)	0.11
Need for postnatal repair at birth (%)	2/23 (9)	8/40 (20)	0.24
Intact motor function (first sacral motor level) at birth (%)	6/23 (26)	28/40 (70)	< 0.01
Motor function at birth (metameric level)	L4 (L1-S1)	S1 (L1-S1)	< 0.01
Clubfeet at birth (%)	13/23 (56)	7/40 (17)	< 0.01
Need for hydrocephalus treatment in the first year of life (%)^c^	6/19 (32)	11/38 (29)	0.84
Independent ambulation (with or without orthotics) at 30 months of age (%)	3/12 (25)	13/26 (50)	0.15
Intact motor function (first sacral motor level) at 12 months of life	4/12 (33)	16/24 (67)	0.06

Large lesion was defined when volume was > 2.7 cc.

L2, second lumbar vertebrae; CSF, cerebrospinal fluid;

^a^Represents the comparisons between the large and non-large lesion group. Quantitative data were expressed as mean ± standard deviation if normal distribution or median (range) if non-normal distribution (as detected by Kolmogorov–Smirnov). Quantitative variables were compared using t-test for independent samples if there was a normal distribution. If a non-normal distribution was present non-parametric tests were used (Mann–Whitney U test). Qualitative variables were compared using Chi-square or Fisher’s exact test. A p-value < 0.05 was considered significant.

^b^Grade 0 (normal); grade 1 (visible fourth ventricle and cisterna magna without cerebellar displacement below the foramen magnum, tentorium could be vertically oriented, and tectal beaking could be present); grade 2 (visible cisterna magna without displacement of cerebellum below the foramen magnum, no visible fourth ventricle); grade 3 (cerebellar ectopia below the foramen magnum and obliteration of all posterior fossa CSF spaces).

^c^Hydrocephalus treatment: including ETV and/or ventriculoperitoneal shunt.

Comparisons between large and non-large MMC lesions in the fetoscopic or the open-repair groups are presented in Supplementary Table 1 and Supplementary Table 2. There was no difference between large and non-large MMC lesions for neuroanatomic findings by MRI at the time of referral and 6 weeks after the prenatal surgery in both subgroups.

At referral, there was no correlation between the volume of the lesion and ventricular size (r = 0.03, p = 0.78), or anatomical level of the lesion (r = 0.18, p = 0.16).

At six post-operative weeks, there was no correlation between the volume of the lesion and hindbrain herniation (HBH) grade (r = 0.04, p = 0.78) or ventricular size (r = 0.1, p = 0.99).

### Impact of the MMC volume on prenatal motor function

Fetuses with large MMC sac volumes showed a higher rate of clubfeet and a lower proportion of intact motor function at the time of referral (Table 1). At the time of referral, a higher MMC volume increases the risk of having clubfeet by 9.5 times (95% CI [2.1–41.8]) and reduces the chances of having an intact motor function by 0.19 times (95% CI [0.1–0.6]) (Table 2).

**Table 2:** Predictors for neurosurgical and neonatal characteristics in cases of large myelomeningocele.

Variables	Logistic regression^a^		
	aOR	95% CI	p
**Pre-operative evaluation**			
Intact motor function^b^	0.19	0.1–0.6	< 0.01
Clubfeet^b^	9.5	2.1–41.8	< 0.01
**Post-operative US evaluation**			
Intact motor function 6 weeks after the surgery^c^	2.3	0.2–28.1	0.51
Intact motor function at last US before delivery^c^	–	–	–
**Neonatal outcomes**			
Intact motor function (first sacral motor function) at birth^d^	0.09	0.01–0.49	< 0.01
Clubfeet at birth^e^	3.7	0.8–18.3	0.11

Large lesion was defined when volume was > 2.7 cc.

aOR, adjusted odds-ration, CI, confidence interval, CSF, cerebrospinal fluid.

^a^Represents the predictive value of large NTD for neurosurgical, post-operative and neonatal outcomes.

^b^Adjusted on high anatomical level of lesion.

^c^Adjusted on the type of repair (fetoscopic or open-hysterotomy), high anatomical level of lesion, learning curve (First 12 cases were considered as learning curve in both types of repair) and intact motor function at referral.

^d^Adjusted on the type of repair (fetoscopic or open-hysterotomy), high anatomical level of lesion, learning curve (first 12 cases were considered as learning curve in both types of repair), gestational age at delivery and intact motor function at referral.

^e^Adjusted on the type of repair (fetoscopic or open-hysterotomy), high anatomical level of lesion, learning curve (first 12 cases were considered as learning curve in both types of repair), gestational age at delivery and presence of clubfeet at referral.

During the last US scan before delivery, the proportion of cases with an intact motor function was higher in cases of non-large lesions compared to large lesions (Table 1).

In the fetoscopic subgroup, there was no difference between large and non-large MMC lesions for the proportion of clubfeet or intact motor function at the time of referral, 6 weeks after the surgery or during the last US before delivery (Supplementary Table 1).

In the open-hysterotomy subgroup, there was a higher proportion of cases with clubfeet and a lower proportion of cases with intact motor function at the time of the referral in the large MMC group compared to the non-large MMC group (Supplementary Table 2).

### Impact of the MMC volume on postnatal neurological short outcomes

At birth, cases with larger MMCs showed a higher rate of clubfeet and a lower proportion of intact motor function compared to non-large MMCs (Table 1). There was a trend for a higher proportion of intact motor function at 12 months of life.

Higher MMC volume reduces the chances of having an intact motor function at birth (OR = 0.11 CI95[0.01–0.49], p < 0.01) after adjusting for the presence of an intact motor function at initial evaluation, anatomical level of the lesion, type of surgery, gestational age at delivery, and learning curve.

Dehiscence or leakage of CSF, need for postnatal repair of the defect at birth, need for hydrocephalus treatment in the first year of life, and independent ambulation at 30 months of age was similar between large and non-large MMC (Table 1).

In the fetoscopic subgroup, there were significantly fewer cases with an intact motor function at birth in the large MMC group compared to the non-large MMC group, and a trend for a higher proportion of cases with clubfeet in the large MMC group (Supplementary Table 1). In this subgroup, having a large MMC reduces the chances of having an intact motor function at birth (OR = 0.1 CI95[0.01–0.6], p = 0.02) after adjusting for the learning curve, anatomical level of the lesion, presence of an intact motor function at referral, and gestational age at delivery.

In the open-hysterotomy subgroup, there were significantly more cases with an intact motor function, and a lower proportion of cases with clubfeet in the non-large MMC group compared to the large MMC group (Supplementary Table 2).

## Discussion

Results from this study suggest that cases considered to be candidates for in-utero MMC repair with large MMC lesions are expected to have an increased odds for having clubfeet at birth (3.7-fold) and are less likely to have an intact motor function (sacral one motor function) at birth (0.09-fold) after a prenatal repair compared to non-large MMC lesions.

Oliver et al. reported their experience with 182 NTD cases prenatally repaired (142 MMC and 40 MS), using the same methodology we used in this study to assess the volume of the lesion with similar volumes (4.7 cc in cases of MMC with clubfeet or 3 cc in cases of MMC without clubfeet)^10^. Their findings are consistent with previous reports of improved postnatal lower extremity function in prenatally repaired cases of myeloschisis compared to MMC^11–13^. A recent study exploring prenatal neurologic findings in cases of open spinal dysraphism found no association between MMC sac volume and prenatal clubfeet^14^. However, comparison with our cohort may be difficult because they did not report the anatomical level of the lesion, which is a well-known predictive factor for clubfeet^12^.

An inverse relationship between sac volume and degree of HBH severity was reported by Nagaraj et al., and it was hypothesized that MMC sac volume may have a protective effect on the degree of HBH by maintaining some hydrostatic pressure within the spinal canal^14^. We only included cases that underwent prenatal repair who had HBH, so we did not compare cases with or without HBH.

Experimental and clinical studies suggest that the neurological deficits associated with NTD occur in a step-wise fashion^6, 15, 16^. Our study confirms previous reports that concluded that larger MMC sizes are associated with lower extremity impairment^10^, an observation that could be described as a “third-hit” hypothesis.

First, NTD results in a closure defect in the posterior portion of the vertebral spine in the third week of gestation^6^. The leak of cerebro spinal fluid (CSF) through the defect exerts a siphoning effect on the cerebellum and medulla. As the gestation proceeds, HBH impairs the path and dynamics of CSF flow resulting in hydrocephalus, which exacerbates the herniation through the foramen magnum, worsening the problem.

Second, the segmental neurological damage at the NTD site can be secondary to both chemical toxicity and recurrent physical trauma of the neural placode in the intra-uterine environment, as reported in animal studies^4, 17, 18^.

Third, the lower extremity impairment that we described in cases of large MMC, may be due to increased traction of the neural placode and nerve roots due to mechanical expansion of the larger sacs. It is possible that the additive effects of all these mechanisms may lead to a more permanent damage of the placode nerve root even after prenatal repair or large MMC.

Our results indicate that the fetal spinal cord and nerves are sensitive to stretch injury which could impact fetal surgical planning for in-utero treatment. When deciding technique and closure strategies for repair, additional care should be shown to minimize traction to the MMC lesion and nerves. Based on our results, measuring the volume of the lesion prior to surgery would be worth considering to assist in intraoperative decision making.

This study included fetuses from two different operative approaches undergoing prenatal repair from a single center. Standard protocols for evaluation and management of care were used for all cases. We acknowledge that the retrospective design is a major limitation of our study. Due to the outcomes seen in NTD cases being multifactorial in nature, it is possible the assumed effect secondary to nerve root overstretching in large lesions could be due to accumulating effects of the volume of the lesion or intrauterine insults that are unknown.

In our cohort, fetuses who present with a large MMC (> 2.7 cc in our cohort) at referral for the evaluation of a prenatal MMC repair are less likely to have an intact motor function at birth and more likely to present clubfeet at birth. These results suggest that spinal cord and nerves are sensitive to stretch injury, a “third-hit” hypothesis.

## Methods

This is a retrospective study of 103 fetuses who underwent a prenatal MMC repair (fetoscopic or open-hysterotomy technique) performed at a single institution between November 2011 and March 2019. All patients who underwent prenatal NTD repair during this period were included in this study. Myeloschisis cases (n = 34), cases where surgery was aborted due to fetal intolerance (n = 3), the lesion was not confirmed to be a myelomeningocele (MMC) by direct visualization (n = 2), or when the mother had a contraindication for an MRI study (n = 1) were excluded from the analysis. All fetuses who underwent prenatal repair and satisfied all MOMS trial inclusion criteria and none of the exclusion criteria were included^9^.

### Initial evaluation

At the time of referral, all patients underwent fetal MRI on a 1.5-T Philips Ingenia superconducting magnet (Philips Medical Systems, Best, The Netherlands). Details of imaging protocol are presented in Supplementary Material. Ventricular width was measured bilaterally at the level of the posterior horns of the lateral ventricles determined by the location of the parieto-occipital fissure on coronal images. Mean ventricular width was calculated, and ventriculomegaly was defined as a mean ventricular width > 10 mm. Severe ventriculomegaly was defined as ≥ 15 mm.

At the time of referral, detailed transabdominal fetal US examinations (General Electric Voluson E8 or E10, GE Healthcare, Milwaukee, WI, USA) were performed in all patients referred to our Fetal Center in order to evaluate patient eligibility for prenatal NTD repair. The anatomical level of the lesion was defined as the upper bony spinal defect on US scans. A high level of the lesion was defined as an upper bony spinal defect at or above the second vertebral body (L2)^9^.

**Figure 1:**
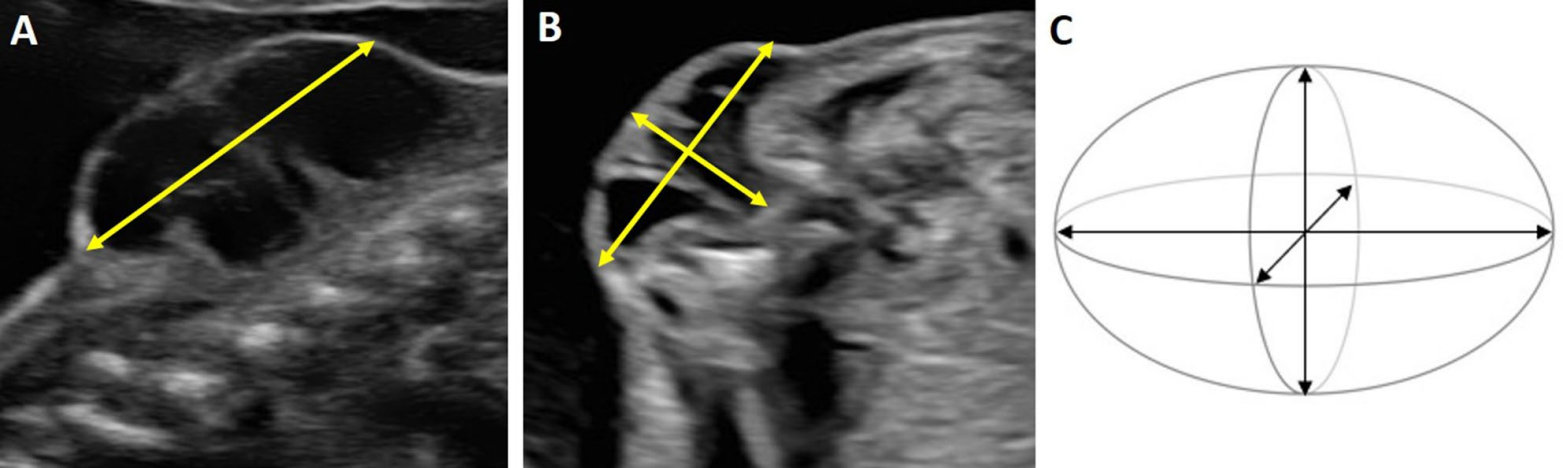
Volume of the myelomeningocele: ultrasound measurements. (**A**) Sagittal view of a myelomeningocele at 20 weeks of gestation: length. (**B**) Transversal view of a myelomeningocele at 20 weeks of gestation: width and depth. (**C**) Volume of the myelomeningocele: prolate ellipsoid volume: length × width × depth × 0.52.

Measurements of the volume of the lesions were performed using US scans to calculate the volume of the prolate ellipsoid (volume = length × width × depth × 0.52)^10^ (Fig. 1).

ROC curve analysis and Youden index were performed to determine the best cut-off to predict an intact motor function at referral based on the volume of the lesion. A 2.7 cc cut-off was found to have the best sensitivity (0.63) and best specificity (0.75) to predict an intact motor function at referral (Supplementary Material). Therefore, a large lesion was defined as greater than 2.7 cc and a non-large lesion as less than or equal to 2.7 cc.

### Prenatal NTD repair

The surgical approach for NTD repair was determined based on maternal choice. During the pre-surgical evaluation, patients were not informed specifically about the volume of the lesion; however, the anatomical level of the lesion was discussed with them. Patients who underwent a prenatal NTD repair were managed per our standard protocol of steroid administration, prophylactic tocolysis (preoperative indomethacin and post-operative nifedipine, and intra- and post-operative magnesium sulfate), and prophylactic antibiotics (cefazolin or if allergic, clindamycin and gentamicin). Open repair was considered the standard of care for prenatal NTD repair and was performed through an open-hysterotomy approach using the same methodology as reported by the MOMS trial^9^. From 2014, fetoscopic repair was offered under our fetoscopic repair protocol provided by the U.S. Food and Drug Administration (FDA) IDE #G140201, the Baylor College of Medicine Institutional Review Board (IRB) protocols H-34680 (7/1/2014) and H-43359 (11/15/2018), our Fetal Therapy Board, and a Data Safety Monitoring Board. These protocols are registered on ClinicalTrials.gov (NCT02230072 and NCT03794011)^19^. The fetoscopic technique was performed as described elsewhere^20^. A single layer closure was performed in the first 23 cases of our MMC cohort. In all subsequent cases, a three layer closure was performed using a collagen patch on top of the dura (Durepair, Medtronic, USA), with muscle flaps and skin closure^21^. During both fetoscopic and open-hysterotomy surgeries, if a complete skin closure was not expected to be possible due to excessive tension on the skin edges, relaxing incisions were performed. These are bilateral vertically oriented incisions performed 2–3 cm from the edge of the defect to facilitate a tension-free midline closure.

Data was retrospectively obtained under an IRB (Baylor College of Medicine) approved protocol H-38479 (2/11/2016). All patients signed an informed consent prior to inclusion in the study. All methods were performed in accordance with the relevant guidelines and regulations. The first 12 cases in both the fetoscopic and the open-hysterotomy techniques, including prenatal repair of myeloschisis, were considered to be part of our learning curve^20^.

### Post-repair evaluation

Six weeks after the repair, the posterior fossa was carefully evaluated by MRI, and the degree of Chiari II malformation was evaluated using Sutton et al.^22^ (Supplementary Material). Reversal of HBH was identified when the most caudal portion of the cerebellum was seen above the foramen magnum. Ventricular width was measured bilaterally at the level of the posterior horns of the lateral ventricles determined by the location of the parieto-occipital fissure on coronal MRI images. Mean ventricular width was calculated, and ventriculomegaly was defined as a mean ventricular width > 10 mm. Severe ventriculomegaly was defined as ≥ 15 mm.

### Evaluation of the prenatal motor function

Ultrasound video clips of the lower extremity movements were reviewed by an expert OB/GYN examiner to score the level of neurologic function based on metameric nerve distribution (Supplementary material) at three different time points: (1) At the time of referral, (2) 6 weeks after prenatal surgical repair and (3) during the last US scan before delivery. Since sacral one (S1) motor function reflects an “intact” motor function, the proportion of cases with S1 motor function was calculated at each of the time points of motor function assessment.

### Management and indications for delivery and labor

Further details about the management of cases who underwent open or fetoscopic NTD repair are included in the Supplementary Material.

### Evaluation at birth

The repair site was clinically examined by a pediatric neurosurgeon within the first 24 h of life to identify any area of dehiscence or CSF leakage at the repair site. The functional segmental motor function was determined by a detailed neurological examination performed by a pediatric neurosurgeon within the first 48 h of life, as reported elsewhere^23^. Details of the neurological examination are included in the Supplementary Material section. Plantar flexion of the ankle was attributed to the first sacral metameric level (S1), which is considered an “intact” motor function.

After discharge, infants were followed at our institution’s multidisciplinary Spina Bifida Clinic, or at their referral centers, every 3 months during their first 12 months of life, and biannually afterward. The need for hydrocephalus treatment was determined using standard clinical and imaging criteria. Ventriculo-peritoneal shunt or endoscopic third ventriculostomy with choroid plexus cauterization was performed if indicated.

Ambulatory skills were evaluated at 30 months of life, and classified as (1) walking independently (with or without orthotics), (2) walking with an assistive device (crutches or walker), or (3) non-ambulatory.

### Statistical analysis

Data analysis was performed using the Statistical Package for Social Sciences (SPSS), version 24.0 (IBM Corporation, Armonk, NY, USA). Quantitative variables were expressed as mean ± standard deviation if they had a normal distribution (by Kolmorov-Smirnov test) and compared with Student’s t-test. Quantitatives variables with non-normal distribution were expressed as median [range] and compared with Mann–Whitney U test. Pearson’s Chi square or Fisher’s exact tests were used to compare qualitative data. Correlations between the volume of the lesion and other quantitative variables such as the anatomical level of the lesion (using a numerical score, Th5 = 1 to S1 = 14), ventricular size, and the HBH grading classification, were performed using Spearman correlation analyses. Linear and logistic regression analyses were performed, controlling for potential confounding variables (anatomical level of lesion, learning curve, type of repair, presence of clubfeet, intact motor function at referral, gestational age at delivery) when indicated. Predictive values of large NTD for neurosurgical, post-operative and neonatal outcomes were expressed as odds ratios (OR) with a 95% confidence interval (95% CI).

## Supplementary Information

Supplementary Information.

## Data Availability

AS (corresponding report) and RC (primary author) have full access to all the data in the study and take responsibility for the integrity of the data and the accuracy of the data analysis. Data could be available on request to the corresponding author.
