# Supplementary material for: Impact of the volume of the myelomeningocele sac on imaging, prenatal neurosurgery and motor outcomes: a retrospective cohort study
Source: Sci Rep. 2021 Jun 23;11:13189. doi: 10.1038/s41598-021-92739-2 (PMC8222266; doi:10.1038/s41598-021-92739-2)
Supplement: Supplementary file 1 — Supplementary Information. [file 41598_2021_92739_MOESM1_ESM.docx]

**Title: Impact of the volume of the myelomeningocele sac on imaging, prenatal neurosurgery and motor outcomes: a retrospective cohort study**

Authors: Romain Corroenne^1^; Amy R. Mehollin-Ray^1,2^; Ms. Rebecca M. Johnson^1^; William E. Whitehead^3^; Jimmy Espinoza^1^; Jonathan Castillo^4^; Heidi Castillo^4^; Gunes Orman^2^; Roopali Donepudi^1^; Thierry A.G.M. Huisman^2^; Ahmed A. Nassr^1^; Michael A. Belfort^1^; Magdalena Sanz Cortes^1^; Alireza A. Shamshirsaz*^1^.

1. Department of Obstetrics and Gynecology, Texas Children’s Hospital & Baylor College of Medicine, Houston, Texas, USA
2. E. B. Singleton Department of Pediatric Radiology, Texas Children’s Hospital & Department of Radiology, Baylor College of Medicine, Houston, Texas, USA
3. Department of Neurosurgery, Texas Children’s Hospital & Baylor College of Medicine, Houston, Texas, USA
4. Department of Pediatrics, Texas Children’s Hospital & Baylor College of Medicine, Houston, Texas, USA

***Corresponding author:**

Alireza A. Shamshirsaz, MD

Division of Fetal Therapy and Surgery

Baylor College of Medicine

Houston TX, 77030

Phone: 832-826-7470

Email: [shamshir@bcm.edu](mailto:shamshir@bcm.edu), [alirezashamshirsaz@yahoo.com](mailto:alirezashamshirsaz@yahoo.com)

**MRI imaging protocol**

At the time of referral, all patients underwent fetal MRI on a 1.5-Tesla Philips Ingenia superconducting magnet (Philips Medical Systems, Best, The Netherlands) using a multi-channel phased array body receive only coil. All studies were performed without fetal or maternal sedation. The standard imaging protocol consisted of multiplanar T2-weighted Half-Fourrier Acquisition Single Shot Turbo Spin Echo (HASTE) imaging, T1-weighted gradient echo imaging, T2-weighted imaging balanced steady-state free precession (bSSFP), and diffusion-weighted imaging (DWI). All MRI studies were interpreted by subspecialty board-certified pediatric radiologists or pediatric neuroradiologists with extensive expertise in fetal MR imaging.

**ROC curve analysis for the prediction of intact motor function at referral based on the volume of the lesion**

Area under the cure = 0.70


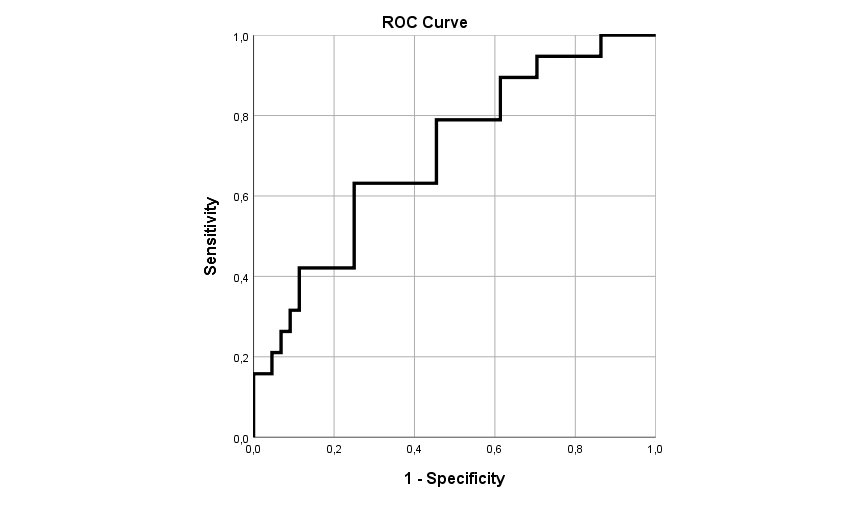


**Chiari II malformation classification**

Six weeks after the repair, the posterior fossa was carefully evaluated by MRI, and the degree of Chiari II malformation was evaluated as: grade 0 (normal); grade 1 (visible fourth ventricle and cisterna magna without cerebellar displacement below the foramen magnum, tentorium could be vertically oriented, and tectal beaking could be present); grade 2 (visible cisterna magna without displacement of cerebellum below the foramen magnum, no visible fourth ventricle); grade 3 (cerebellar ectopia below the foramen magnum and obliteration of all posterior fossa CSF spaces).

**Prenatal ultrasounds assessment of the motor function according to the lower extremity movements**

| **Metameric Level** | **Function** |
| --- | --- |
| L1-L2 | Hip Flexion/adduction |
| L3 | Knee extension |
| L4-L5 | Knee flexion and/or dorsal flexion of the ankle |
| S1 | Plantar flexion of ankle (gas pedal) |

**Management and indications for delivery and labor**

Cases who underwent a fetoscopic repair were scheduled for induction of labor at 39-40 weeks applying standard obstetrical principles or scheduled for elective cesarean delivery at 39 weeks if indicated (breech, previous cesarean deliveries and lack of maternal intent to have vaginal birth after cesarean or fetal macrocephaly)^22,23^. Open-hysterotomy repair cases were scheduled for cesarean delivery at 37 weeks. If there were any obstetrical indications for delivery prior to 37 weeks, it would also be performed by C-section.

^1^Belfort MA, Whitehead WE, Shamshirsaz AA, Bateni ZH, Olutoye OO, Olutoye OA, Mann DG, Espinoza J, Williams E, Lee TC, Keswani SG, Ayres N, Cassady CI, Mehollin-Ray AR, Sanz Cortes M, Carreras E, Peiro JL, Ruano R, Cass DL. Fetoscopic Open Neural Tube Defect Repair. Obstet Gynecol. 2017;129(4):734-743. doi:10.1097/AOG.0000000000001941.

^2^Kohn JR, Rao V, Sellner AA, Sharhan D, Espinoza J, Shamshirsaz AA, Whitehead WE, Belfort MA, Sanz Cortes M. Management of Labor and Delivery After Fetoscopic Repair of an Open Neural Tube Defect. Obstet Gynecol. 2018;131(6):1062-1068. doi:10.1097/AOG.0000000000002577.

**Neurological evaluation of motor function at birth**

The neurological examination including assessment of sensory, motor and cutaneous level of each lower extremity was obtained at each clinic visit. Motor levels were indicated by the lowest myotomes involved in active motor function. If different neurological levels were observed in each lower extremity from the same subject, the better segmental level observed was the one considered for analysis^1^.

^1^Kellogg R, Lee P, Deibert CP, Tempel Z, Zwagerman NT, Bonfield CM, Johnson S, Greene S. Twenty years’ experience with myelomeningocele management at a single institution: lessons learned. *J Neurosurg Pediatr*. October 2018:439-443. doi:10.3171/2018.5.PEDS17584.

**Supplementary Table 1. Comparison of neurological, neurosurgical and neonatal outcomes between large and non-large myelomeningocele in case of fetoscopic repair**

|  | **Large lesions**  **(n=14)** | **Non-large lesions**  **(n=23)** | **p^a^** |
| --- | --- | --- | --- |
| **Pre-operative MRI evaluation** |  |  |  |
| Gestational age at the time of referral (weeks) | 24.3 (19.3-25.5) | 24.1 (20.3-25.7) | 0.70 |
| Volume of the lesion (cc) | 4.5 (3-44.5) | 1.3 (0.05-2.4) | <0.01 |
| Anatomical level of the lesion ≥ L2 (%) | 5/14 (36) | 5/23 (22) | 0.35 |
| Mean size of bilateral posterior ventricular horns at referral (mm) | 11.2 (7-18) | 12 (6-18) | 0.57 |
| Ventriculomegaly defined as mean of posterior horn width >10mm (%) | 9/14 (64) | 17/23 (74) | 0.53 |
| Severe ventriculomegaly defined as mean of posterior horn width >15mm (%) | 2/14 (14) | 5/23 (22) | 0.68 |
| Clubfeet at referral (at least one foot, %) | 6/14 (43) | 4/23 (17) | 0.09 |
| **Pre-operative US motor function evaluation** |  |  |  |
| Intact motor function (first sacral level) at the time of referral (%)  Motor function at the time of referral (metameric level) | 7/14 (50)  L4 (L1-S1) | 17/23 (74)  S1 (L1-S1) | 0.14  0.17 |
| **Surgery** |  |  |  |
| Gestation age at the time of surgery (weeks) | 25.3 (24.4-25.9) | 24.8 (22.9-26) | 0.04 |
| Interval between time of pre-operative MRI and surgery (weeks) | 1 (0.1-6.5) | 0.5 (0.1-4.2) | 0.57 |
| Duration of surgery (min) | 259 (133-394) | 230 (163-356) | 0.30 |
| Need for relaxing incisions (%) | 3/14 (21) | 1/23 (4) | 0.14 |
| **Post-operative MRI evaluation** |  |  |  |
| Gestational age of post-operative MRI (weeks) | 30.7 (28-33) | 31 (28-32) | 0.67 |
| Hindbrain herniation severity^b^ | 1 (0-3) | 1 (0-3) | 0.33 |
| Reversal of hindbrain herniation after surgery (%) | 6/12 (50) | 17/22 (77) | 0.10 |
| Ventricle size after surgery (mm) | 15.5 (10-26.5) | 16.5 (7-23.5) | 1 |
| Ventriculomegaly defined as mean of posterior horn width >10mm (%) | 12/12 (100) | 19/22 (86) | 0.54 |
| Severe ventriculomegaly defined as mean of posterior horn width >15mm (%) | 5/12 (58) | 16/22 (73) | 0.39 |
| **Post-operative US motor function evaluation** |  |  |  |
| Intact motor function 6 weeks after the surgery (%)  Motor function 6 weeks after the surgery (metameric level) | 6/11 (54)  L4 (L1-S1) | 12/19 (63)  S1 (L1-S1) | 0.64  0.42 |
| Intact motor function at last US scan before delivery (%)  Motor function at last US scan before delivery (metameric level) | 2/6 (33)  L4 (L1-S1) | 13/18 (72)  S1 (L1-S1) | 0.15  0.25 |
| **Neonatal outcomes** |  |  |  |
| Gestational age at delivery (weeks) | 38.1 (26-40) | 37.7 (29.9-40.6) | 0.49 |
| Female gender (%) | 4/14 (29) | 11/23 (48) | 0.25 |
| Birth weight (grams) | 2735 (870-3745) | 2920 (1375-4430) | 0.39 |
| Dehiscence or leakage of CSF at birth (%) | 2/14 (14) | 6/23 (26) | 0.68 |
| Need for postnatal repair at birth (%) | 2/14 (14) | 5/23 (22) | 0.69 |
| Intact motor function (first sacral motor level) at birth (%)  Motor function at birth (metameric level) | 4/14 (29)  L4 (L1-S1) | 17/23 (74)  S1 (L3-S1) | <0.01  <0.01 |
| Clubfeet at birth (%) | 7/14 (50) | 5/23 (22) | 0.07 |
| Need for hydrocephalus treatment in the first year of life (%)^c^ | 6/12 (50) | 7/21 (33) | 0.35 |
| Independent ambulation (with or without orthotics) at 30 months of age (%) | 0/7 (0) | 6/13 (46) | 0.05 |
| Intact motor function (first sacral motor level) at 12 months | 2/6 (33) | 8/12 (67) | 0.32 |

Abbreviations: L2, second lumbar vertebrae; CSF, cerebrospinal fluid;

Large lesion was defined when volume was > 2.7 cc.

a-Represents the comparisons between the large and non-large lesion group. Quantitative data were expressed as mean ± standard deviation if normal distribution or median (range) if non-normal distribution (as detected by Kolmogorov-Smirnov). Quantitative variables were compared using t-test for independent samples if there was a normal distribution. If a non-normal distribution was present non-parametric tests were used (Mann-Whitney U test). Qualitative variables were compared using Chi-square or Fisher’s exact test. A p-value <0.05 was considered significant.

b- grade 0 (normal); grade 1 (visible fourth ventricle and cisterna magna without cerebellar displacement below the foramen magnum, tentorium could be vertically oriented, and tectal beaking could be present); grade 2 (visible cisterna magna without displacement of cerebellum below the foramen magnum, no visible fourth ventricle); grade 3 (cerebellar ectopia below the foramen magnum and obliteration of all posterior fossa CSF spaces).

c- Hydrocephalus treatment: including ETV and/or ventriculoperitoneal shunt. **Supplementary Table 2. Comparison of neurological, neurosurgical and neonatal outcomes between large and non-large myelomeningocele in case of open-hysterotomy repair**

|  | **Large lesions**  **(n=9)** | **Non-large lesions**  **(n=17)** | **p^a^** |
| --- | --- | --- | --- |
| **Pre-operative MRI evaluation** |  |  |  |
| Gestational age at the time of referral (weeks) | 22.3 (18.7-24.5) | 22.2 (18-24.5) | 0.56 |
| Volume of the lesion (cc) | 4.5 (3-7.2) | 0.9 (0.1-2.3) | <0.01 |
| Anatomical level of the lesion ≥ L2 (%) | 2/9 (22) | 3/17 (18) | 1 |
| Mean size of bilateral posterior ventricular horns at referral (mm) | 11 (8-16.5) | 10.5 (6-16) | 0.34 |
| Ventriculomegaly defined as mean of posterior horn width >10mm (%) | 7/9 (78) | 9/17 (53) | 0.22 |
| Severe ventriculomegaly defined as mean of posterior horn width >15mm (%) | 3/9 (33) | 4/17 (23) | 0.66 |
| Clubfeet at referral (at least one foot, %) | 4/9 (44) | 0/17 (0) | <0.01 |
| **Pre-operative US motor function evaluation** |  |  |  |
| Intact motor function (first sacral level) at the time of referral (%)  Motor function at the time of referral (metameric level) | 4/9 (44)  L4 (L1-S1) | 16/17 (94)  S1 (L1-S1) | 0.01  0.04 |
| **Surgery** |  |  |  |
| Gestation age at the time of surgery (weeks) | 25.3 (24-25.9) | 24.1 (21.3-25.9) | 0.04 |
| Interval between time of pre-operative MRI and surgery (weeks) | 2.9 (0.6-7) | 2.4 (0.1-7.9) | 0.60 |
| Duration of surgery (min) | 157 (129-160) | 150 (94-180) | 0.77 |
| Need for relaxing incisions (%) | 0/9 (0) | 3/17 (18) | 0.53 |
| **Post-operative MRI evaluation** |  |  |  |
| Gestational age of post-operative MRI (weeks) | 29.3 (24-33) | 29.5 (27-35) | 0.97 |
| Hindbrain herniation severity^b^ | 1 (0-2) | 1 (1-3) | 0.19 |
| Reversal of hindbrain herniation after surgery (%) | 7/7 (100) | 11/13 (85) | 0.52 |
| Ventricle size after surgery (mm) | 14 (12.5-26) | 15 (6-21) | 0.73 |
| Ventriculomegaly defined as mean of posterior horn width >10mm (%) | 7/7 (100) | 13/15 (87) | 1 |
| Severe ventriculomegaly defined as mean of posterior horn width >15mm (%) | 3/7 (43) | 8/15 (53) | 1 |
| **Post-operative US motor function evaluation** |  |  |  |
| Intact motor function 6 weeks after the surgery (%)  Motor function 6 weeks after the surgery (metameric level) | 3/8 (37)  L1 (L1-S1) | 12/16 (75)  S1 (L1-S1) | 0.07  0.08 |
| Intact motor function at last US scan before delivery (%)  Motor function at last US scan before delivery (metameric level) | 3/6 (50)  L4 (L1-S1) | 6/6 (100)  S1 (L1-S1) | 0.09  0.18 |
| **Neonatal outcomes** |  |  |  |
| Gestational age at delivery (weeks) | 36 (29.6-37) | 34.6 (27.3-37.1) | 0.90 |
| Female gender (%) | 3/9 (33) | 10/17 (59) | 0.41 |
| Birth weight (grams) | 2505 (1114-3095) | 2570 (624-3175) | 1 |
| Dehiscence or leakage of CSF at birth (%) | 0/9 (0) | 4/17 (23) | 0.26 |
| Need for postnatal repair at birth (%) | 0/9 (0) | 3/17 (18) | 0.53 |
| Intact motor function (first sacral motor level) at birth (%)  Motor function at birth (metameric level) | 2/9 (22)  L4 (L1-S1) | 11/17 (64.7)  S1 (L1-S1) | 0.04  0.05 |
| Clubfeet at birth (%) | 6/9 (67) | 2/17 (12) | <0.01 |
| Need for hydrocephalus treatment in the first year of life (%)^c^ | 0/7 (0) | 4/17 (23) | 0.28 |
| Independent ambulation (with or without orthotics) at 30 months of age (%) | 3/5 (60) | 7/13 (54) | 1 |
| Intact motor function (first sacral motor level) at 12 months of life | 2/6 (33) | 8/12 (67) | 0.32 |

Abbreviations: L2, second lumbar vertebrae; CSF, cerebrospinal fluid;

Large lesion was defined when volume was > 2.7 cc.

a-Represents the comparisons between the large and non-large lesion group. Quantitative data were expressed as mean ± standard deviation if normal distribution or median (range) if non-normal distribution (as detected by Kolmogorov-Smirnov). Quantitative variables were compared using t-test for independent samples if there was a normal distribution. If a non-normal distribution was present non-parametric tests were used (Mann-Whitney U test). Qualitative variables were compared using Chi-square or Fisher’s exact test. A p-value <0.05 was considered significant.

b- grade 0 (normal); grade 1 (visible fourth ventricle and cisterna magna without cerebellar displacement below the foramen magnum, tentorium could be vertically oriented, and tectal beaking could be present); grade 2 (visible cisterna magna without displacement of cerebellum below the foramen magnum, no visible fourth ventricle); grade 3 (cerebellar ectopia below the foramen magnum and obliteration of all posterior fossa CSF spaces).

c-Hydrocephalus treatment: including ETV and/or ventriculoperitoneal shunt.
